# The assessment of ischaemic burden: validation of a "functional" jeopardy score against cardiovascular magnetic resonance

**DOI:** 10.1186/1532-429X-15-S1-O108

**Published:** 2013-01-30

**Authors:** Shazia T Hussain, Geraint Morton, Kalpa De Silva, Roy Jogiya, Mark Peterzan, Andreas Schuster, Matthias Paul, Amedeo Chiribiri, Divaka Perera, Eike Nagel

**Affiliations:** 1 Imaging sciences, Kings College, London, UK; 2 Cardiovascular Dept, Kings College, London, UK; 3 Cardiology Dept, London Chest Hospital, London, UK

## Background

Due to the increasing emphasis on guiding patient management and revascularisation based on the extent rather than just the presence of ischaemia it is important to understand the relationship between parameters obtained in the catheter laboratory, such as luminal coronary artery stenosis, fractional flow reserve (FFR) and the extent of the ischaemic burden. Classic jeopardy scores estimate the area of myocardium at risk based on luminal stenosis severity without integrating their haemodynamic significance, whereas FFR assesses the impact of a stenosis on flow but is not influenced by the volume of subtended myocardium. Incorporating FFR measurements to a jeopardy score to produce a "functional jeopardy score" may provide a rapid method in the catheterisation laboratory to estimate ischaemic burden. The objective of this study is to assess the relationship between classical anatomical jeopardy scores, functional jeopardy scores (based on the combination of anatomical and haemodynamic data) and the extent of ischaemia identified on cardiovascular magnetic resonance (CMR) perfusion imaging.

## Methods

39 patients with angina and known or suspected coronary artery disease (CAD) referred for coronary angiography prospectively underwent high-resolution CMR perfusion imaging and coronary angiography. Fractional Flow Reserve (FFR) was measured in all vessels with a stenosis of >50%. The APPROACH and BCIS-1 jeopardy scores were calculated based purely on angiographic anatomy using a cut off of both 50% (APP50 and BCIS50) and 70% (APP70 and BCIS70) as well as after integration of FFR (APPFFR and BCISFFR) and compared with the extent of ischaemia identified on CMR perfusion imaging.

## Results

**Figure 1:**
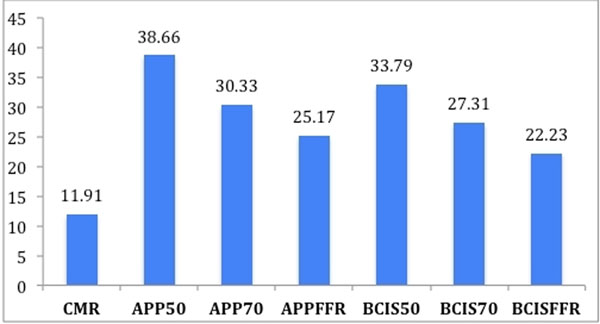
Mean ischaemic Burden Estimation Mean values (percentage) of ischaemic burden measured by CMR and angiographic jeopardy scores (APPROACH and BCIS-1) based on 50 % and 70% QCA thresholds and with incorporation of the FFR data (BCISFFR and APPFFR). The BCIS-1 score has been converted into a percentage for comparative purposes. CMR = cardiovascular magnetic resonance FFR=fractional flow reserve

The correlation between the extent of ischaemia measured by CMR and the anatomical jeopardy score based on a 50 % threshold was moderate (APP50 r=0.56 p=0.0002; BCIS50 r=0.47, p=0.0012) see table1. This was improved by using the 70% threshold (APP70 r=0.69 p=0.0001, BCIS70 r=0.67 p=0.0001). Integration of FFR data resulted in good correlation (APPFFR r=0.76, p=0.0001, BCISFFR r=0.78, p=0.0001.The extent of ischaemia measured by the different scores is demonstrated in Fig 1. Bland Altman analysis reveals an overestimation of the area at risk with all anatomical scoring. APP50 and APP70 with a mean bias of 27.6% and 19.2 respectively. BCIS50 and BCIS70 score of 27.7%. This improved to 14.1% and 11.1% with the functional APPFFR and BCISFFR scores respectively and was associated with smaller confidence intervals.

**Table 1:** Mean values of angiographic scores and correlation with CMR

Score	Mean result (SD)	Correlation with CMR (r value	Correlation with CMR (r value	Mean bias
APP50	38.66 (26)	0.60	p<0.05	27.6
APP70	30.33 (27.8)	0.69	p<0.05	19.2
APPFFR	25.17 (23.4)	0.76	p<0.05	14.1
BCIS50	4 (3.1)	0.47	p<0.05	27.7
BCIS70	3.27 (3.17)	0.67	p<0.05	16.2
BCISFFR	2.67 (2.7)	0/78	p<0.05	11.1

## Conclusions

Anatomical and functional jeopardy scores overestimate the extent of ischaemia, when compared to CMR. Integrating physiological information from FFR to angiographic lesion characterization to generate a functional jeopardy score improves the estimation of ischaemic burden in the catheterization laboratory.

## Funding

This work was supported by a European Union Grant (Grant number 224495 to GM, EN); the British Heart Foundation (Research Excellence Award RE/08/003 and FS/10/029/28253 to AS, DP, EN); the Biomedical Research Centre (grant number BRC-CTF 196 to AS, DP, EN) and the Wellcome Trust and EPSRC (grant number WT 088641/Z/09/Z to AC, EN).

